# Revolutionizing thyroid nodule diagnosis in Hashimoto’s thyroiditis: AI-driven radiomics and deep learning model

**DOI:** 10.1097/JS9.0000000000004546

**Published:** 2026-03-05

**Authors:** Fan Wu, Ting Pan, Xuanwei Huang, Kaiyuan Huang, Jingjing Shi, Linlin Mao, Yeqin Ni, Dingcun Luo, Yu Zhang

**Affiliations:** a Department of Oncological Surgery, Affiliated Hangzhou First People’s Hospital, Westlake University School of Medicine, Hangzhou, Zhejiang, China; b Cancer Center, Department of Pathology, Zhejiang Provincial People’s Hospital (Affiliated People’s Hospital), Hangzhou Medical College, Hangzhou, Zhejiang, China; c The Fourth Clinical Medical College, Zhejiang Chinese Medical University, Hangzhou, Zhejiang, China

**Keywords:** artificial intelligence, deep learning, Hashimoto’s thyroiditis, papillary thyroid carcinoma, SHAP value

## Abstract

**Objective:**

Accurately distinguishing between benign and malignant thyroid nodules (TNs) in the context of Hashimoto’s thyroiditis (HT) is challenging. This study aimed to explore the diagnostic efficacy of artificial intelligence models constructed using radiomics and deep learning (DL) features extracted from the ultrasound images of TNs in the setting of HT. The study also aimed to quantitatively compare the diagnostic performance of these models against that of fine-needle aspiration (FNA) cytology combined with BRAFV600E gene mutation testing so as to establish a superior diagnostic paradigm for TNs in HT.

**Methods:**

We analyzed the clinical data and preoperative ultrasound images of 1585 patients with HT admitted to eight hospitals in China between 1 January 2018, and 30 December 2023. Radiomics features were extracted from each manually annotated and standardized region of interest in the images. The DL features based on various convolutional neural network models were also extracted, including 11 pre-trained DL models based on real images and the image features extracted using a combined DL–radiomics (DLR) approach. Least absolute shrinkage and selection operator regression was used to select features with nonzero coefficients. Further, eight machine learning methods were employed to construct prediction models, namely the DLR models, incorporating both DL and radiomics features. The importance of features in contributing to the model was prioritized using the SHapley Additive exPlanations (SHAP) method for interpretation. The predictive performance of the models was evaluated using the area under the receiver operating characteristic curve (AUC), accuracy, sensitivity, and specificity. Two rounds of reader studies were conducted (first round: independent reading; second round: guided reading) to validate the clinical application value of the DLR model, with results compared with FNA biopsy outcomes.

**Results:**

A total of 1561 radiomics features and 256 DL features were extracted from the original images. The DLR model, leveraging the ResNet152 neural network, could effectively differentiate between benign and malignant nodules in the context of HT. The AUC, accuracy, sensitivity, and specificity of the logistic regression (LR) model, based on these features, were 0.917 [95% confidence interval (CI): 0.838–0.988], 85.7%, 81.8%, and 86.6%, respectively, in the validation cohort and 0.827(95% CI: 0.777–0.876), 93.9%, 80.9%, and 96.3%, respectively, in the external test cohort. The SHAP summary plot illustrated how feature values influenced their impact on the model, whereas the SHAP force plot showed the integrated impact of features on individual responses. Gradient-weighted class activation mapping (Grad-CAM)-generated heatmaps from DL models visually highlighted high-risk areas in HT. The DLR model outperformed five junior-level physicians in terms of diagnostic accuracy, sensitivity, and specificity in the validation cohort. The diagnostic performance of all clinicians was significantly enhanced with the assistance of the DLR model, with no statistical difference detected compared with the FNA biopsy results.

**Conclusions:**

The DLR model combined with the SHAP and Grad-CAM method can improve the diagnostic performance of radiologists in identifying benign and malignant TNs in the context of HT. The diagnostic efficacy of this visualization model is comparable to that of FNA cytology combined with gene mutation testing. The DLR model can enhance the diagnostic ability of radiologists in differentiating between benign and malignant TNs in the context of HT, thereby minimizing unnecessary biopsies. Additionally, it can aid clinicians in making personalized decisions regarding the necessity of biopsy or even surgery by providing intuitive visual explanations.

## Introduction

Hashimoto’s thyroiditis (HT) is the most prevalent autoimmune thyroid disease^[1]^, with a higher prevalence in women than men^[2]^. The histological hallmarks of HT include lymphocytic infiltration and follicular destruction, leading to progressive atrophy and fibrosis of thyroid tissue^[1]^. HT frequently coexists with thyroid nodules (TNs) and exhibits an increased susceptibility to thyroid cancer^[3,4]^. The conventional sonographic features of HT often manifest as diffuse lesions accompanied by numerous inflammatory pseudo-nodules, altering the sonographic landscape of TNs^[5]^. Studies report inconsistent characteristics of malignant nodules against the diffuse background of HT; some studies suggest smooth margins with varying degrees of calcification in malignant nodules within HT^[6]^, whereas others indicate irregular margins with microcalcifications^[7]^. The diversity of malignant characteristics contributes to greater ambiguity in diagnosis, thereby complicating clinicians’ decision-making processes, increasing the diagnostic challenge, and concurrently augmenting the risks of both misdiagnosis and missed diagnosis. Current evidence indicates significantly lower diagnostic accuracy of both conventional ultrasound and fine-needle aspiration (FNA) for differentiation between benign and malignant TNs in HT compared with non-HT counterparts^[8,10]^. Additionally, FNA is an invasive, more expensive procedure, and the elevated levels of antibodies associated with HT can impair the accuracy of cytological diagnosis and genetic testing. Therefore, novel and effective methods need to be urgently developed to diagnose the benignity or malignancy of TNs in the context of HT.

Researchers have explored novel image features and classification algorithms to enhance the accuracy of ultrasound diagnosis for TNs^[11]^. For instance, Zhao *et al* introduced a local and global feature network for classifying TNs as benign or malignant, achieving an accuracy rate of 89.33%^[12]^. Recently, radiomics based on ultrasound image analysis has demonstrated superior performance compared with other traditional methods^[13]^. Radiomics enables the automatic extraction of a vast number of quantitative image features from medical images, which are often difficult to discern visually^[14,15]^. With the rapid development of radiomics and deep learning (DL) technologies, TN diagnostic systems based on DL and combined DLR approach have gradually become research hotspots. Radiomics is a method using large-scale imaging data for analysis and mining. It can extract features and recognize patterns from imaging data, thereby assisting doctors in early disease diagnosis and prediction. Meanwhile, DL is used to construct deep neural network models to automatically learn and extract high-level features from imaging data for accurate classification and diagnosis. Compared with traditional methods, DLR-based diagnosis of TNs in the context of HT offers significant advantages. First, DL-based methods can automatically learn and extract features from imaging data, thus avoiding the subjectivity and limitations of manual feature design. Second, DLR-based diagnostic systems have strong generalization capabilities, which enable them to adapt to different TN image data. This implies that the system can be applied across different medical institutions and imaging equipment, providing doctors with more reliable and consistent diagnostic results. Furthermore, DLR-based diagnostic systems facilitate more comprehensive and detailed nodule feature analysis, aiding doctors in making more accurate diagnoses and predictions. Artificial intelligence (AI) can improve the diagnostic accuracy of less experienced or nonspecialist clinicians^[16,17]^. DL methods do not require human experts to predefine features beforehand^[18]^. They can automatically extract multi-layer features that clinicians may not recognize. Previous studies have reported high diagnostic performance of AI in diagnosing TNs without HT^[19]^. However, few studies have used DLR to diagnose the benignity or malignancy of TNs in the context of HT, and the results of such studies have been unsatisfactory^[20,21]^.

**HIGHLIGHTS**

- Innovative AI Model for Thyroid Nodule Diagnosis in Hashimoto’s Thyroiditis: This study introduces a novel diagnostic approach using an artificial intelligence (AI) model that combines radiomics and deep learning (DL) features extracted from ultrasound images. This model is specifically designed to accurately distinguish between benign and malignant thyroid nodules (TNs) in patients with Hashimoto’s thyroiditis (HT).
- Robust Predictive Performance: The developed deep learning–radiomics (DLR) model, leveraging the ResNet152 neural network, demonstrated exceptional predictive performance with an AUC of 0.917, accuracy of 85.7%, sensitivity of 81.8%, and specificity of 86.6% in the training cohort. These results underscore the model’s effectiveness in differentiating TNs.
- Feature Importance Analysis Using SHAP Method: To gain insights into the model’s decision-making process, the SHapley Additive exPlanations (SHAP) method was employed. This analysis revealed the importance of individual features in contributing to the model’s predictions, providing valuable insights for understanding and interpreting the AI model’s performance.
- Clinical Validation and Enhancement of Diagnostic Accuracy: The DLR model was clinically validated through two rounds of reader studies, demonstrating its superiority in diagnostic accuracy, sensitivity, and specificity compared to five junior-level physicians. Furthermore, with the assistance of the DLR model, the diagnostic performance of all clinicians was significantly enhanced, approaching the gold standard of fine-needle aspiration biopsy results. This model has the potential to reduce unnecessary biopsies and aid in guiding personalized treatment strategies for patients with HT.

Numerous radiomics models have yielded promising results in limited datasets or applications^[22,23]^. However, their clinical practicality is hindered by unclear or difficult-to-interpret features within the internal mechanisms of the models^[24]^. The SHapley Additive exPlanation (SHAP) method offers a solution to this issue. SHAP provides a consistent and fair explanation of feature importance by assessing the contribution of each feature to the predictions of the model^[25]^. The combination of SHAP and radiomics allows for an interpretable illustration of the model^[26,27]^, thus elucidating the importance of features in the predictive model and their contributions to individual predictions. Gradient-weighted class activation mapping (Grad-CAM) is an interpretability technique for convolutional neural networks that generates gradient-weighted heatmaps to visualize crucial regions in model decision-making^[28]^. When analyzing ultrasound images, this approach pinpoints lesion-focused areas prioritized by DL models, offering intuitive visual diagnostic evidence.

This study is novel insofar as it is the first to delineate regions of interest in ultrasound images to differentiate benign from malignant TNs. We used more than1000 samples across 9 centers to develop a specialized diagnostic model for distinguishing nodule malignancy in the context of HT. Further, we employed the SHAP method to interpret and visualize the model, thereby aiding in enhancing the diagnostic efficacy of clinicians. While developing and validating our AI-based model, transparency in the reporting of artificial intelligence guidelines was followed to ensure the transparency and reliability of our findings^[29]^.

## Method

### Study population

**Figure 1.**
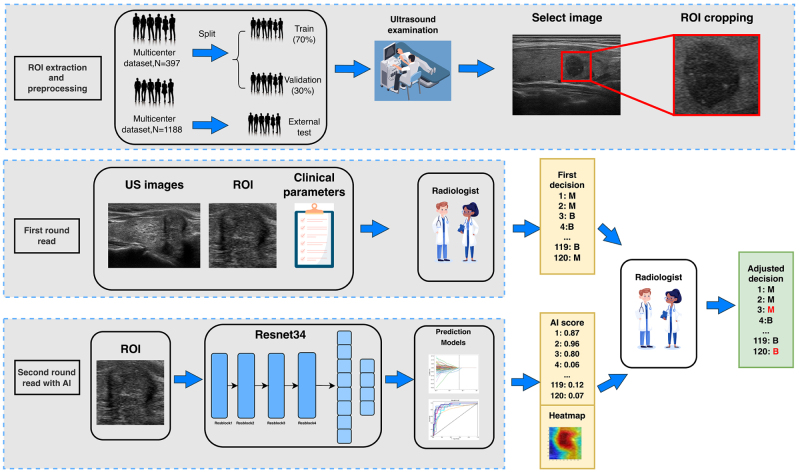
Workflow scheme of the radiomics and deep learning analysis.

We collected training and validation datasets from patients with HT to establish and evaluate a predictive model for differentiating benign from malignant TNs in the context of HT. The study workflow is illustrated in Figure 1. Five-fold cross-validation was employed to split the development dataset into a training set (70%) and a validation set (30%) to determine optimal model parameters during model development. The dataset was collected between January 2018 and December 2023, encompassing data from two imaging centers: Hospital A and Hospital B. Two ultrasonographers with 5 years of experience reviewed the ultrasound images of all datasets, excluding those with low image quality, artifacts, or discordant interpretations among the reviewers. The consensus-read dataset comprised 397 patients from the 2 hospitals (Hospital A, *n* = 357; Hospital B, *n* = 40). A total of 1188 patients were enrolled from 8 collaborating hospitals in China. Further details are provided in Supplemental Digital Content Methods S1, available at: http://links.lww.com/JS9/G420.

### Inclusion and exclusion criteria

The inclusion criteria were as follows: (1) patients who underwent thyroid ultrasonography with images retained prior to surgery; (2) patients with a postoperative pathological diagnosis of HT; (3) patients who had undergone FNA biopsy of TNs in the past; and (4) patients with complete clinical and pathological information. The exclusion criteria were as follows: (1) patients with uncontrolled subacute thyroiditis or hyperthyroidism; (2) patients with a history of thyroid radiotherapy or thyroid surgery; (3) unsatisfactory image quality; and (4) tumors with a maximum diameter greater than 5 cm or a minimum diameter less than 3 mm. This study was approved by the ethics committee of Hangzhou First People’s Hospital, and written informed consent was obtained from all patients. Supplemental Digital Content Figure S1, available at: http://links.lww.com/JS9/G420 presents the specific inclusion and exclusion screening process.

### Image acquisition and FNA diagnosis of TNs

The diagnostic equipment used for thyroid examinations included the Mylab 70 XVG and Mylab color Doppler ultrasound systems, with probe frequencies ranging from 7 to 13 MHz. Prior to surgery, the patients were placed in the supine position to ensure full exposure of the neck. The physician identified suspicious malignant nodules and then interpreted and documented the two-dimensional ultrasound image characteristics of the nodules. Two experienced ultrasonographers assessed preoperative tumor information using ultrasound, without prior knowledge of the pathological results of the TNs or the results of the DL-based risk assessment. Besides the ultrasound images, each patient’s dataset included a lesion dataset solely for detecting the presence of tumor lesions, as described later. Detailed descriptions of the FNA and BRAFV600E mutation analysis processes are presented in Supplemental Digital Content Methods S2 and S3, available at: http://links.lww.com/JS9/G420, respectively.

### Image preparation and correlation coefficients test

Thyroid ultrasonography was conducted on patients within 1 week prior to surgery. Two ultrasonographers, each with at least 5 years of experience in ultrasound diagnosis of thyroid diseases, independently performed manual segmentation of the ultrasound images. The ITK-SNAP software was used to manually delineate the region of interest (ROI) along the tumor edges on each image (http://www.itksnap.org). A *Z*-score normalization method was employed to standardize the ultrasound images, achieving a standard normal distribution of image intensities. The reproducibility and reliability of selected features were assessed employing intra-class correlation coefficients among radiologists. For complete methodological details, refer to Supplemental Digital Content Methods S2, available at: http://links.lww.com/JS9/G420 and Supplemental Digital Content Table S1, available at: http://links.lww.com/JS9/G420.

### Radiomics and DL feature extraction

We used the Pyradiomics open-source library (version 3.0.1)^[30]^ to extract 2D radiomics features from the ultrasound images. The extracted features encompassed the following: (1) 14 shape parameters, including tumor area and maximum diameter; and (2) 1547 texture features, including 306 first-order features, 272 gray-level size zone matrix features, 85 neighboring gray tone difference matrix features, 272 gray-level run-length matrix features, 374 gray-level co-occurrence matrix features, and 238 gray-level dependence matrix features. A total of 1561 features were extracted from each ultrasound image, and all features were normalized to a uniform range from zero to one.

We developed a DL model using postoperative pathology as the ground truth to assess the probability of benignity versus malignancy of TNs in ultrasound images. Also, we used pre-trained models such as resNet18, resNet34, resNet50, resNet101, resNet152, densenet121, densenet161, densenet169, densenet201, inception_v3, and manasnet0_5 as the foundational architectures to extract DL features from each normalized ROI image. The model was trained using the stochastic gradient descent optimizer with an initial learning rate of 0.01, decayed using the cosine annealing algorithm for 50 epochs, with a batch size of 32. We used the fixed network parameters after model training as feature extractors. More specifically, we used the output of the penultimate layer of the trained convolutional neural network (CNN) to define DL features. The DL extracted 1536 features from the ROIs of the three-phase images for each patient.

### Gradient-weighted class activation mapping

Subsequently, we stored the model with the best performance, evaluated using the area under the receiver operating characteristic (ROC) curve (AUC) on the validation set. We used the Grad-CAM algorithm to display heatmaps, which is a tool designed to comprehend and interpret the decision-making process of DL models when processing images or other data. Grad-CAM integrates the model’s gradient information with activation maps, thus visually demonstrating how the model predicts a given input sample to belong to a certain class across various regions. It employs the backpropagation algorithm to compute the gradients of each neuronal activation in the model relative to the input sample, with these gradients weighted to reflect the model’s attention to the image regions embedded within that neuron. The weighted gradients are then used to recreate images representing the model’s focal areas, thus resulting in a visual decision map. This image helps illustrate how the model combines various features to make predictions when processing specific inputs. This methodology is invaluable for comprehending and interpreting the behavior of DL models, especially in scenarios requiring the explanation of complex model decision-making processes. It facilitates improved model design and tuning and enhances the understanding of DL principles in educational and other contexts. We applied Grad-CAM to the final convolutional layer of the model to generate heatmaps with sufficient spatial resolution^[31]^.

### Model explanation and visualization

SHAP is rooted in cooperative game theory, with the aim of dismantling the black box effect of machine learning models. This enables the identification and prioritization of features that determine complex classifications. The SHAP values represent the average expected marginal contribution of a player in the model, considering all possible coalitions. They are primarily used in scenarios where contributions among players are unequal but the collective efforts yield a benefit^[25]^. We employed the SHAP kernel explainer to evaluate feature attribution, treating each patient’s feature as a player in the game, where the prediction constituted the benefit and each player’s contribution indicated the feature’s importance to the output.

### Construction and analysis of the DLR model

This study constructed a DLR model combining DL and radiomics to further increase the AUC. Initially, each image in the dataset was processed using the model. The activations of the second-to-last fully connected neural network layer were stored and used as the DL features of the image. These DL features (*n* = 256) were concatenated with radiomics features (*n* = 1561) to create a set of combined DLR features (*n* = 1817) for each image. Multiple models were trained with different parameters, and their performance was evaluated using a validation set (measured using the average AUC through five-fold cross-validation). The least absolute shrinkage and selection operator (LASSO) regression algorithm was employed to select the most significant features for distinguishing benign from malignant cases. Using postoperative pathology as the ground truth, we constructed predictive models by inputting the selected Rad, DL, and DLR feature sets into classifiers, which ultimately output the probability of benign or malignant outcomes. The optimal classifier was determined using the development dataset (split into 70% training and 30% validation) to develop and evaluate eight different classifiers: logistic regression (LR), naive Bayes, support vector machine (SVM), K-nearest neighbors (KNN), LightGBM, gradient boosting, AdaBoost, and multi-layer perceptron (MLP). The classifier that produced the best AUC (i.e., the highest average and lowest standard deviation in five-fold cross-validation) was selected for developing the final model. Detailed performance metrics for various classifiers are presented in Supplemental Digital Content Tables S2–S4, available at: http://links.lww.com/JS9/G420. The model performance was assessed based on the confusion matrix (Supplemental Digital Content Figure S3, available at: http://links.lww.com/JS9/G420) and six indicative parameters: accuracy, sensitivity, specificity, positive predictive value (PPV), negative predictive value (NPV), and F1 score of the post-training model. The ROC curves and the AUC were used to evaluate the performance of the three individual models and the ensemble model (Supplemental Digital Content Table S6, available at: http://links.lww.com/JS9/G420). The decision curve analysis (DCA) was conducted to assess the clinical utility of the models by quantifying the net benefit at different threshold probabilities. The predictive results for each sample, along with their corresponding true outcomes, were plotted to demonstrate the distribution of benign and malignant predictions using the classification model.

### Two-round reader study

**Figure 2.**
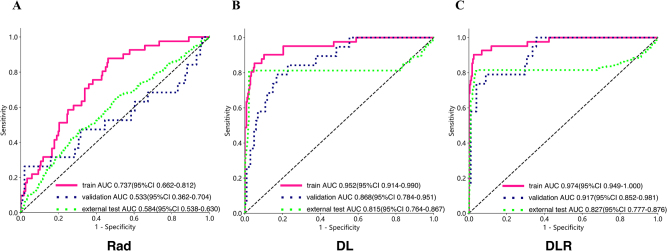
Receiver operating characteristic curves of the three models.

To demonstrate the clinical utility of the DLR model, we conducted two rounds of reader studies to investigate the actual clinical benefits gained by clinicians with the assistance of the DLR model (Fig. 2). Five clinicians with an average of 2 years (range: 1–3 years) of ultrasound experience and another five with an average of 8 years (range: 6–15 years) of experience participated in this study. A total of 120 lesions (including 19 benign ones) from the validation cohort were presented in random order. The clinicians were blinded to each other, the original diagnostic reports, and the final pathological results throughout the process. Detailed information on the two rounds of reader studies is provided in Supplemental Digital Content Methods S6, available at: http://links.lww.com/JS9/G420.

Performance of the three models for predicting thyroid nodules in all datasets. Receiver operating characteristic curves of the (A) radiomics (Rad) dataset, (B) deep learning (DL) dataset, (C) deep learning–radiomics (DLR) dataset.

### Statistical and data analyses

Statistical analyses were conducted using SPSS (version 23.0; IBM Corp., NY, USA) and Python (version 3.10, accessible at https://www.python.org/). The patient characteristics were summarized as numbers (percentages) for categorical variables and means (standard deviations) for continuous variables. The continuous variables were compared between groups using either the two-sided *t* test or the Mann–Whitney *U* test. The Pearson chi-square test was employed for comparing categorical data. A *P* value less than 0.05 indicated a statistically significant difference. LASSO regression analysis and Z-score normalization were performed using Python. Predictive parameters, including accuracy, sensitivity, specificity, PPV, and NPV, were also used to evaluate performance. This work has been reported in line with the REMARK criteria^[32]^.

## Results

### Baseline characteristics

As shown in Table 1, 1595 patients were enrolled in the study. Table 1 summarizes the clinical and conventional ultrasound characteristics of patients in the training, validation, and external test cohorts. No differences were observed in clinical and ultrasound characteristics between the three cohorts (*P* > 0.05). The postoperative pathological results revealed 1347 malignant and 248 benign lesions. This study strictly adhered to the inclusion and exclusion criteria. A detailed screening process is outlined in Supplemental Digital Content Figure S1, available at: http://links.lww.com/JS9/G420. Supplemental Digital Content Table S2, available at: http://links.lww.com/JS9/G420 presents the clinical factors included in the study. Most visually obvious and commonly used ultrasound evaluation factors were excluded from subsequent analyses due to a lack of statistical significance (P > 0.05).

**Table 1:** The comparison of ultrasound clinical features of PTC.

Characteristic	Training set (277)			Validation set (120)			External test set (1198)		
	Malignant	Benign	*P*-value	Malignant	Benign	*P*-value	Malignant	Benign	P-value
Age(years)	45.01 ± 12.20	53.76 ± 9.79	<0.001	42.65 ± 11.63	48.89 ± 5.99	0.025	45.50 ± 12.03	45.44 ± 11.75	0.844
Gender			1			0.442			0.916
Female	210(88.98)	37(90.24)		93(92.08)	19(100.00)		913(90.40)	171(90.96)	
Male	26(11.02)	4(9.76)		8(7.92)	Null		97(9.60)	17(9.04)	
Tumor diameter (mm)	7.77 ± 5.00	8.85 ± 4.32	0.325	8.34 ± 4.25	8.26 ± 5.76	0.468	8.05 ± 4.78	8.12 ± 4.79	0.885
Primary site			0.506			0.371			0.546
Left lobe	96(40.68)	18(43.90)		54(53.47)	13(68.42)		455(45.05)	91(48.40)	
Right lobe	135(57.20)	21(51.22)		42(41.58)	6(31.58)		522(51.68)	93(49.47)	
Isthmus	5(2.12)	2(4.88)		5(4.95)	Null		33(3.27)	4(2.13)	
Location			0.121			0.618			0.202
Up	56(23.73)	11(26.83)		22(21.78)	6(31.58)		248(24.55)	43(22.87)	
Middle	127(53.81)	15(36.59)		44(43.56)	7(36.84)		475(47.03)	104(55.32)	
Down	48(20.34)	13(31.71)		30(29.70)	6(31.58)		254(25.15)	37(19.68)	
Isthmus	5(2.12)	2(4.88)		5(4.95)	Null		33(3.27)	4(2.13)	
Unilateral			0.111			0.344			0.856
Unilateral	199(84.32)	39(95.12)		84(83.17)	18(94.74)		862(85.35)	162(86.17)	
Bilateral	37(15.68)	2(4.88)		17(16.83)	1(5.26)		148(14.65)	26(13.83)	
Multifocality			0.002			0.13			1
Single	185(78.39)	41(100.00)		77(76.24)	18(94.74)		816(80.79)	152(80.85)	
Multifocal	51(21.61)	Null		24(23.76)	1(5.26)		194(19.21)	36(19.15)	
Echogenicity			0.089			0.248			0.145
Isoechoic	9(3.81)	4(9.76)		3(2.97)	2(10.53)		41(4.06)	13(6.91)	
Hypoechoic	226(95.76)	36(87.80)		95(94.06)	17(89.47)		955(94.55)	174(92.55)	
Hyperechoic	1(0.42)	1(2.44)		3(2.97)	Null		14(1.39)	1(0.53)	
Blood flow			0.758			0.547			0.198
None	97(41.10)	18(43.90)		35(34.65)	8(42.11)		397(39.31)	81(43.09)	
Little	12(5.08)	3(7.32)		5(4.95)	Null		47(4.65)	13(6.91)	
Rich	127(53.81)	20(48.78)		61(60.40)	11(57.89)		566(56.04)	94(50.00)	
Vertical and horizontal diameter ratio			0.235			0.948			
≤1	67(28.39)	16(39.02)		28(27.72)	6(31.58)		299(29.60)	54(28.72)	0.876
<1	169(71.61)	25(60.98)		73(72.28)	13(68.42)		711(70.40)	134(71.28)	
Margin			0.136			1			0.121
Smooth	83(35.17)	20(48.78)		32(31.68)	6(31.58)		369(36.53)	57(30.32)	
Spiculated/microlobulated	153(64.83)	21(51.22)		69(68.32)	13(68.42)		641(63.47)	131(69.68)	
Calcification			0.171			0.32			0.921
Rim calcification	2(0.85)	1(2.44)		1(0.99)	1(5.26)		13(1.29)	2(1.06)	
Microcalcification	102(43.22)	10(24.39)		44(43.56)	5(26.32)		404(40.00)	82(43.62)	
Macrocalcification	22(9.32)	6(14.63)		11(10.89)	4(21.05)		111(10.99)	19(10.11)	
Mixed calcification	2(0.85)	Null		2(1.98)	Null		10(0.99)	2(1.06)	
None	108(45.76)	24(58.54)		43(42.57)	9(47.37)		472(46.73)	83(44.15)	

### Radiomics, DL, and construction and validation of the DLR model

We employed various machine learning classifiers, including LR, naive Bayes, SVM, KNN, LightGBM, gradient boosting, AdaBoost, and MLP, to train and validate diagnostic models that predicted the postoperative pathological outcome of TNs in the context of HT on both training and validation sets. Among all classifiers, the LR classifier exhibited relatively better performance with a higher AUC value in the validation set. The radiomics study screened nine features and constructed a model with an AUC of 0.680 (95% CI: 0.568–0.792) (Fig. 2), indicating that effective prediction solely relying on radiomics was challenging. An AUC of 0.584 was achieved in the test cohort. As shown in Tables 2 and Supplemental Digital Content Table S3, available at: http://links.lww.com/JS9/G420, the DL model pre-trained on ResNet152 demonstrated the best performance in diagnosing nodules, with an AUC of 0.886 (95% CI: 0.812–0.960) in the validation set. Additionally, the test cohort achieved an AUC of 0.815 (Brier score = 0.146). Similarly, as presented in Tables 3 and Supplemental Digital Content Table S4, available at: http://links.lww.com/JS9/G420, the DLR model pre-trained on ResNet152 displayed optimal performance in diagnosing nodules, with an AUC of 0.913 (95% CI: 0.838–0.988) in the validation set and 0.827 (95% CI: 0.777–0.876) in the test set (Brier score = 0.139).

**Table 2:** The difference between various DL models.

Model	Groups	Accuracy	AUC	(95%CI)	Sensitivity	Specificity	PPV	NPV
Resnet18	Training	0.996	1	1.000–1.000	0.979	1	1	0.996
	Validation	0.748	0.775	0.667–0.882	0.682	0.763	0.395	0.914
	Test	0.530	0.563	0.517–0.610	0.633	0.511	0.194	0.882
Resnet34	Training	0.921	0.987	0.976–0.998	0.957	0.914	0.692	0.991
	Validation	0.697	0.771	0.666–0.877	0.818	0.67	0.36	0.942
	Test	0.401	0.511	0.469–0.554	0.723	0.341	0.170	0.869
Resnet50	Training	0.832	0.922	0.882–0.962	0.894	0.819	0.5	0.974
	Validation	0.681	0.87	0.798–0.943	0.955	0.619	0.362	0.984
	Test	0.681	0.613	0.5694–0.6571	0.452	0.724	0.234	0.876
Resnet101	Training	0.882	0.976	0.960–0.991	0.979	0.862	0.59	0.995
	Validation	0.765	0.811	0.706–0.916	0.818	0.753	0.429	0.948
	Test	0.614	0.593	0.5502–0.6360	0.574	0.622	0.220	0.887
Resnet152	Training	0.95	0.995	0.990–1.000	0.957	0.948	0.789	0.991
	Validation	0.882	0.886	0.812–0.960	0.818	0.897	0.643	0.956
	Test	0.952	0.815	0.7639–0.8669	0.809	0.978	0.874	0.965
Densenet121	Training	0.935	0.979	0.965–0.994	0.936	0.935	0.746	0.986
	Validation	0.714	0.73	0.612–0.849	0.682	0.722	0.357	0.909
	Test	0.565	0.635	0.5908–0.6786	0.681	0.544	0.217	0.901
Densenet161	Training	0.935	0.979	0.961–0.998	0.915	0.94	0.754	0.982
	Validation	0.588	0.746	0.6470–0.844	0.864	0.526	0.292	0.944
	Test	0.609	0.591	0.5452–0.6374	0.548	0.620	0.211	0.880
Densenet169	Training	0.946	0.99	0.982–0.999	0.957	0.944	0.776	0.991
	Validation	0.739	0.845	0.759–0.932	0.864	0.711	0.404	0.958
	Test	0.529	0.547	0.5017–0.5913	0.559	0.524	0.179	0.864
Densenet201	Training	0.982	0.999	0.996–1.000	0.979	0.983	0.92	0.996
	Validation	0.857	0.855	0.755–0.956	0.682	0.897	0.6	0.926
	Test	0.689	0.578	0.5319–0.6233	0.388	0.746	0.221	0.868
Inception_v3	Training	0.935	0.982	0.969–0.995	0.936	0.935	0.746	0.986
	Validation	0.866	0.864	0.782–0.946	0.636	0.918	0.636	0.918
	Test	0.321	0.544	0.4997–0.5888	0.878	0.218	0.173	0.905
Mnasnet0_5	Training	0.839	0.882	0.830–0.935	0.809	0.845	0.514	0.956
	Validation	0.773	0.629	0.494–0.764	0.364	0.866	0.381	0.857
	Test	0.690	0.524	0.4794–0.5690	0.293	0.764	0.188	0.853

**Table 3:** The difference between various deep learning–radiomics (DLR) models.

Model	Groups	Accuracy	AUC	(95%CI)	Sensitivity	Specificity	PPV	NPV
Resnet18	Training	0.996	1	1.000–1.000	0.979	1	1	0.996
	Validation	0.731	0.820	0.725–0.915	0.864	0.701	0.396	0.958
	Test	0.484	0.591	0.545–0.636	0.723	0.440	0.194	0.895
Resnet34	Training	0.907	0.958	0.928–0.987	0.872	0.914	0.672	0.972
	Validation	0.815	0.798	0.700–0.896	0.636	0.856	0.5	0.912
	Test	0.553	0.569	0.525–0.614	0.590	0.547	0.195	0.878
Resnet50	Training	0.957	0.977	0.959–0.996	0.894	0.97	0.857	0.978
	Validation	0.765	0.837	0.754–0.921	0.818	0.753	0.429	0.948
	Test	0.703	0.577	0.532–0.623	0.356	0.767	0.222	0.865
Resnet101	Training	0.978	0.998	0.996–1.000	0.957	0.983	0.918	0.991
	Validation	0.647	0.762	0.643–0.882	0.773	0.619	0.315	0.923
	Test	0.603	0.607	0.565–0.651	0.606	0.602	0.221	0.891
Resnet152	Training	0.978	0.999	0.998–1.000	0.979	0.978	0.902	0.996
	Validation	0.857	0.913	0.838–0.988	0.818	0.866	0.581	0.955
	Test	0.939	0.827	0.777–0.876	0.809	0.963	0.804	0.964
Densenet121	Training	0.889	0.965	0.946–0.984	0.936	0.879	0.611	0.986
	Validation	0.697	0.819	0.740–0.897	0.909	0.649	0.37	0.969
	Test	0.450	0.567	0.525–0.609	0.734	0.397	0.185	0.889
Densenet161	Training	0.982	0.992	0.984–1.000	0.915	0.996	0.977	0.983
	Validation	0.546	0.739	0.636–0.843	0.909	0.464	0.278	0.957
	Test	0.792	0.607	0.561–0.653	0.293	0.885	0.322	0.870
Densenet169	Training	0.989	0.999	0.997–1.000	0.979	0.991	0.958	0.996
	Validation	0.824	0.818	0.720–0.916	0.591	0.876	0.52	0.904
	Test	0.551	0.589	0.545–0.634	0.617	0.539	0.199	0.883
Densenet201	Training	0.989	0.999	0.998–1.000	0.979	0.991	0.958	0.996
	Validation	0.857	0.901	0.812–0.990	0.864	0.856	0.576	0.965
	Test	0.653	0.593	0.547–0.640	0.505	0.680	0.227	0.881
Inception_v3	Training	0.975	0.999	0.997–1.000	0.979	0.974	0.885	0.996
	Validation	0.689	0.811	0.722–0.899	0.818	0.66	0.353	0.941
	Test	0.563	0.591	0.547–0.635	0.606	0.554	0.202	0.883
Mnasnet0_5	Training	0.821	0.891	0.840–0.941	0.83	0.819	0.481	0.96
	Validation	0.697	0.701	0.586–0.817	0.545	0.732	0.316	0.877
	Test	0.628	0.594	0.550–0.637	0.500	0.651	0.211	0.875

Detailed methods for training the DLR model are provided in Supplemental Digital Content Methods S5, available at: http://links.lww.com/JS9/G420. The comprehensive process of constructing the radiomics, DL, and DLR models, along with feature selection, is outlined in Supplemental Digital Content Table S5, available at: http://links.lww.com/JS9/G420 and Supplemental Digital Content Figure S2, available at: http://links.lww.com/JS9/G420. The confusion matrix allowed for the calculation of evaluation metrics such as accuracy, precision, recall, and F1 score to assess model performance (Supplemental Digital Content Tables S2–S4, available at: http://links.lww.com/JS9/G420). The histogram illustrated the prediction distribution, where the height of each bar corresponded to the number of samples predicted to belong to that category, thus helping to identify any prediction bias in the model (Supplemental Digital Content Figure S4, available at: http://links.lww.com/JS9/G420). The histogram analysis revealed that most samples were correctly classified, but some categories had lower prediction accuracy, potentially indicating overfitting or underfitting of the model on these categories. DCA was used to evaluate the classification results on the validation dataset (Supplemental Digital Content Figure S5, available at: http://links.lww.com/JS9/G420). It provided a comprehensive view of model performance by considering net benefits across various decision thresholds. The calibration curves in Supplemental Digital Content Figure S6, available at: http://links.lww.com/JS9/G420 reveal excellent alignment between the combined model’s predictions and observed outcomes, thereby demonstrating superior fit over individual models. The calibration accuracy was evaluated using the Brier score, where scores of 0–0.25 denoted excellent predictive performance (Supplemental Digital Content Table S8, available at: http://links.lww.com/JS9/G420). Supplemental Digital Content Table S7, available at: http://links.lww.com/JS9/G420 compares the AUC, accuracy, sensitivity, and specificity of the three models in the training, validation, and test sets.

### Model interpretation and visualization

**Figure 3.**
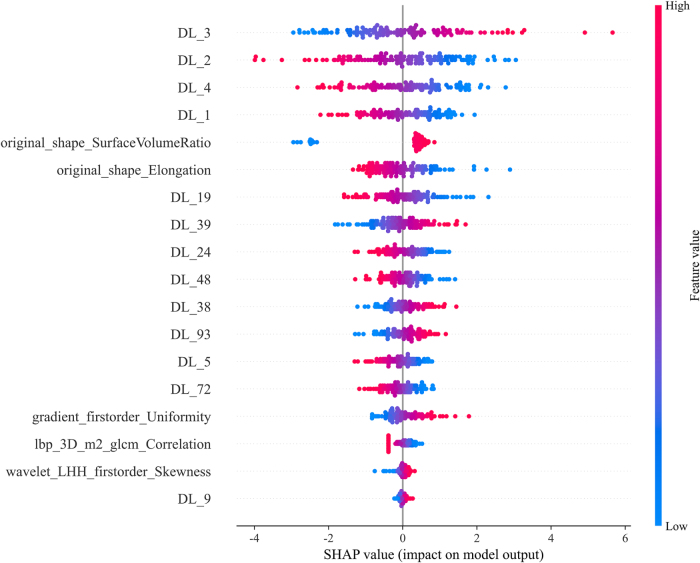
SHAP summary plots of Deep learning-radiomics model.

**Figure 4.**
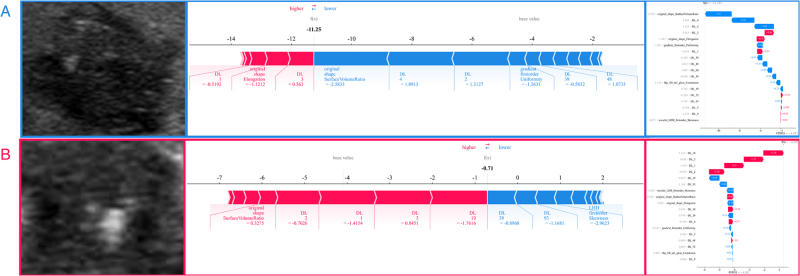
SHAP force plots.

SHAP provides quantitative explanations for the LR model methodology. The SHAP summary plot offers a visually concise graphic by representing the range and distribution of feature importance to the model’s output and linking feature values to feature impacts. Features are first sorted by their global importance. Each point represents the SHAP value of each feature from a patient, plotted horizontally and stacked vertically to reflect the density of the same SHAP value. Each point is colored according to the feature value, ranging from low (blue) to high (red). As shown in Figure 3, DL-3 was the most important feature in distinguishing between benign and malignant classifications. The density of DL-3 showed variations in the SHAP values of this feature across the cohort. The coloring indicated that the model output increased with a decrease in the feature value. SHAP force plots (Fig. 4) can explain assessments for individual patients. They visualize the SHAP values of features as forces that increase or decrease the assessment, with each prediction starting from a base value – the average SHAP value across all predictions. The length of the arrows illustrates the degree of contribution (as a percentage) of a specific feature to the SHAP value. The color of the arrows indicates whether the contribution is positive (red) or negative (blue).

The plot illustrated the feature relevance and combined feature attributions to the model’s predictive performance.

GLCM, local binary patterns; LBP, local binary patterns; SHAP, SHapley Additive exPlanations.

SHAP force plots explained how the radiomics-clinical model discriminates the benign and malignant nodules in two cases. The nodules of case A were benign, and the nodules of case B were malignant. For instance, high feature DL-4 contributed to the increase in the assessment probability of the benign group. Case B had a DL-2 value of −1.4154, while a lower DL-2 value of patient A (1.3127) contributed to assessing the benign group.

GLCM, local binary patterns; LBP, local binary patterns; SHAP, SHapley Additive exPlanations.

Meanwhile, Grad-CAM is a visualization technique that generates class activation maps (CAMs) by analyzing the feature maps and gradients of the last convolutional layer in CNNs. These heatmaps visually demonstrate the image regions the model focuses on when making diagnostic decisions. In the heatmaps, the blue regions highlighted in malignant cases are significantly larger than those in benign cases, with most of these regions located within the lesion. In contrast, the red regions in the heatmaps of benign cases mainly focus on the interior of the lesion. In our study, Grad-CAM revealed the most sensitive features used by the model to identify TNs, such as the edge, shape, and internal structure of the nodules. The doctors use the heatmaps generated using Grad-CAM to intuitively see the areas that the model considers most influential to the diagnostic outcome (Fig. 5). This visualization not only helps doctors understand the decision-making logic of the model but also enhances their trust in the diagnostic results of the model. Furthermore, when the model’s predictions are inconsistent with doctors’ clinical judgments, the visual explanations provided by Grad-CAM can serve as a basis for further discussion and analysis.

**Figure 5a.**
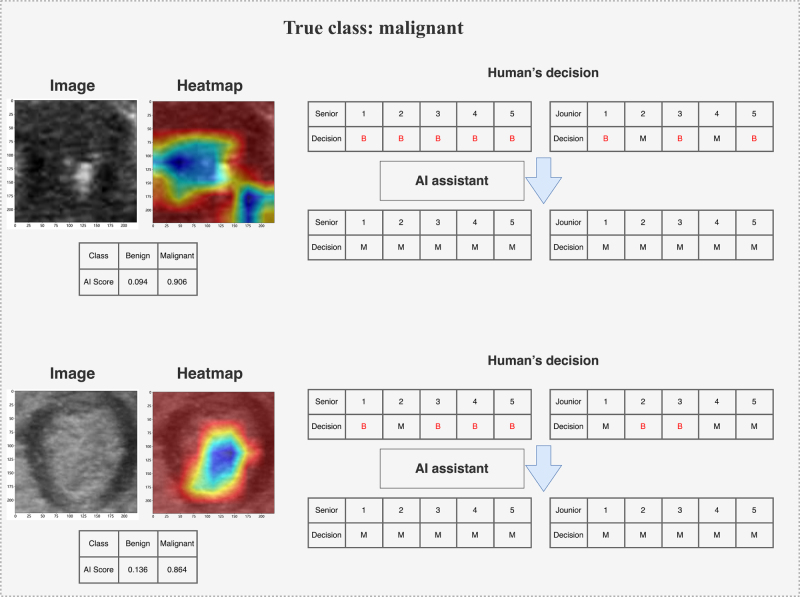
Typical cases of our DLR model guiding radiologists to make correct decisions.

DLR, deep learning–radiomics.

**Figure 5b.**
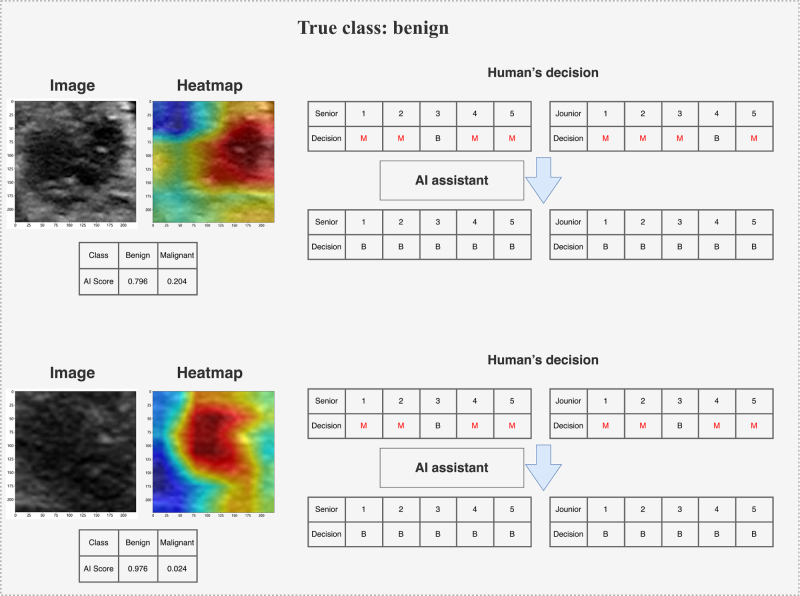
Typical cases of our DLR model guiding radiologists to make correct decisions. DLR, deep learning–radiomics.

### AI-assisted diagnosis

**Figure 6.**
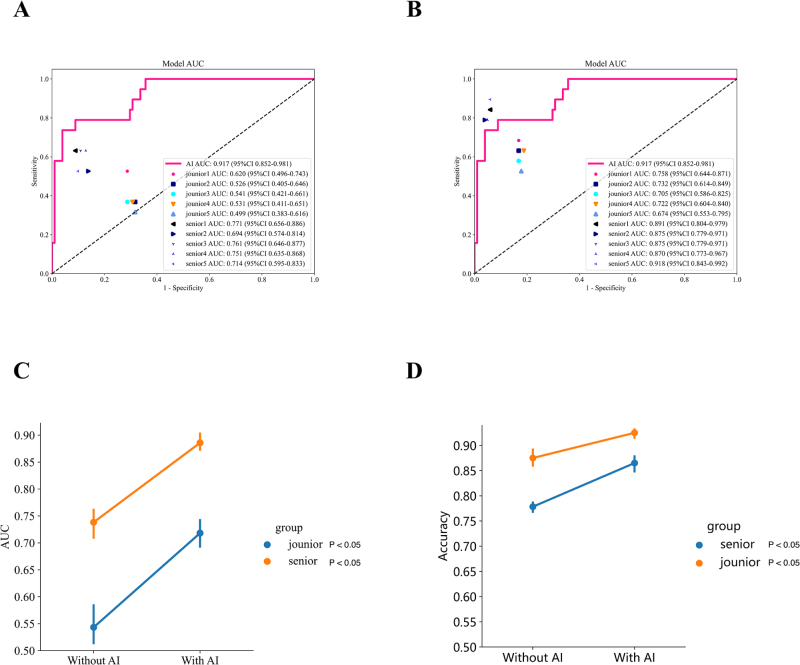
Decision-making of radiologists before and after AI assistance. Figure A compares the predictive performance between junior and senior doctors versus the model in the initial year; Figure B contrasts the predictive performance among junior and senior doctors versus the model after model-assisted diagnosis; Figure C illustrates the changes in AUC for junior and senior doctors before and after model assistance; Figure D depicts the trends in ACC for junior and senior doctors before and after model assistance.

AUC, area under the curve; ACC, accuracy.

The decisions made by the first round of radiologists were compared with those of the DLR model. The ROC curve of the DLR model, the diagnostic results of each radiologist, and the average diagnostic results of all radiologists of varying seniority levels are shown in Figure 6A and 6B. Ten sonographers were recruited for this study, including five with an average of 2 years (range: 1–3 years) of ultrasound experience and five with an average of 8 years (range: 6–15 years) of experience. The diagnostic results of these 10 clinicians were compared with those of the model. Our study clearly showed that the diagnostic performance of senior radiologists was comparable to, or even better than, that of the DLR model. In contrast, junior radiologists demonstrated inferior diagnostic performance compared with the model, with very few reaching the upper left region of the ROC curve. This suggested that our model generally outperformed junior radiologists.

In a two-round reader study, we analyzed diagnostic changes made by five junior and five senior clinicians before and after AI assistance. In the internal validation cohort, all clinicians exhibited improved accuracy and specificity with AI assistance. To more vividly illustrate the clinical value of our DLR model, Figure 5 displays instances where clinicians changed their initial decisions after receiving AI input. Detailed changes before and after AI assistance for clinicians are provided in Supplemental Digital Content Table S7, available at: http://links.lww.com/JS9/G420. Although the AI scores and heatmaps provided by the DLR model could sometimes mislead clinicians’ decisions in certain cases, the overall diagnostic performance of all 10 clinicians in the validation cohort displayed a significant improvement trend before and after DLR assistance. In the validation cohort, the DLR model had a positive impact in terms of improving the average diagnostic performance AUC and accuracy of radiologists (Fig. 6). Additionally, Supplemental Digital Content Figure S7, available at: http://links.lww.com/JS9/G420, presents the approximate changes in sensitivity and specificity for the 10 clinicians with and without AI assistance.

Figure 5 showcases typical cases where the DLR model guided radiologists to make correct decisions. The upper images display two TNs. Most radiologists initially considered these lesions to be benign but achieved elevated accuracy after obtaining additional information generated by the DLR model. In these two cases, the DLR model showed significant differences in scores for benign versus malignant lesions. The lower images show two benign lesions. Most radiologists initially misclassified these lesions as malignant but achieved elevated accuracy after obtaining additional information from the DLR model. In these two cases, redder regions in the heatmaps indicate a higher likelihood of benign lesions, whereas blue regions indicate a higher likelihood of malignancy. These regions are primarily located within the ROI image, consistent with radiologists’ diagnostic patterns for benign and malignant lesions. The heatmaps generated using Grad-CAM for model visualization exhibited different patterns in images of benign versus malignant nodules^[33]^. More specifically, the red regions in malignant cases were larger than those in benign cases and were mostly located outside the lesions. In contrast, the red regions in the heatmaps of benign lesions were mainly distributed within the images. Furthermore, the radiologists noted that, for malignant lesions, the blue regions in the heatmaps were primarily located within the tumor, such as in calcified or hypoechoic areas. Figure 6 summarizes the total scores given by 10 radiologists for each lesion in the validation cohort with and without DLR model assistance. Figure 6A and 6B displays the AUC diagnostic performance without and with DLR model assistance, respectively. Ultimately, all radiologists systematically improved their AUC for diagnosing benign and malignant lesions with the help of the DLR model (Fig. 6C), and a systematic increase in accuracy (Figure 6D), specificity, and, particularly, sensitivity was noted (Supplemental Digital Content Figure S6, available at: http://links.lww.com/JS9/G420).

### Comparative diagnostic performance of the DLR model and FNA-based methods

In the validation cohort, we calculated and compared the diagnostic efficacy of different methods using metrics including AUC, accuracy (ACC), sensitivity (SEN), and specificity (SPE), as summarized in Table 4. All metrics were derived based on the postoperative pathological gold standard. The FNA combined method integrated both cytopathological examination and BRAFV600E mutation analysis. Although the ACC of our proposed DLR model was slightly lower than that of the FNA combined method, it achieved a higher AUC. Making a complete diagnosis based solely on clinical experience proved challenging for both junior and senior radiologists. However, with the assistance of the DLR model, all clinicians exhibited significant improvements in ACC, SEN, SPE, and AUC. Notably, the diagnostic performance of senior radiologists aided by AI was comparable to that of radiologists using the FNA combined method, suggesting that AI-assisted diagnosis may achieve a level of accuracy similar to that of invasive biopsy.

**Table 4:** Diagnostic efficacies of different methods in the validation cohort.

Method	ACC	SENS	SPEC	AUC	PPV	NPV
Clinicians only						
Junior	0.648	0.389	0.697	0.543	0.194	0.859
Senior	0.84	0.738	0.887	0.738	0.498	0.920
DLR model	0.917	0.737	0.950	0.917	0.737	0.950
Clinicians with DLR model assistance						
Junior	0.792	0.611	0.826	0.718	0.397	0.919
Senior	0.930	0.821	0.950	0.886	0.759	0.966
FNA combined	0.942	0.842	0.960	0.901	0.800	0.970

ACC, accuracy; NPV, negative predictive value; PPV, positive predictive value; SENS, sensitivity; SPEC, specificity.

## Discussion

This large-scale multicenter study demonstrated that an ultrasound-based radiomics model integrated with DL, combined with visualization, effectively differentiated between benign and malignant TNs in the context of HT. When applied to assist clinicians, this model significantly enhanced diagnostic performance while substantially reducing the necessity for invasive procedures. Our approach provides a promising, noninvasive, practical, and reliable strategy for evaluating nodule malignancy in the context of HT.

First, this study pioneered the integration of radiomics with DL models to differentiate between benign and malignant TNs in the context of HT. Radiomics is a methodology exploring feature significance through analysis and mining of large-scale imaging data, displaying immense potential in the early diagnosis and prediction of diseases. However, radiomics studies alone have limitations in certain scenarios. For instance, Cao *et al*^[11]^ examined differentiated thyroid cancer and nodules and showed that, although radiomics features can provide valuable diagnostic information, the diagnostic performance of the model is limited by sample size and the accuracy of feature selection. Moreover, the process of extracting radiomics features is complex, and most features are manually designed, thus restricting the reproducibility and generalization ability of the model. This study also found that a model solely relying on radiomics features (AUC = 0.680) failed to achieve ideal diagnostic performance. DL constructs deep neural network models to automatically learn and extract high-level features from imaging data, thus enabling accurate classification and diagnosis. However, DL methods alone also present challenges. Li *et al*^[19]^ used a deep CNN model to diagnose thyroid ultrasound images and achieved high diagnostic accuracy. However, the model’s interpretability was poor, being regarded as a “black box” operation. Furthermore, the performance of DL models highly depends on the quantity and quality of training data, and the generalization ability of the model may be limited for datasets with small sample sizes or complex backgrounds. In this study, although we used a ResNet-based DL model for feature extraction, we faced the issue of insufficient model interpretability. Some studies have begun to explore the combination of both to overcome the limitations of using radiomics or DL alone. However, these studies still have deficiencies in certain aspects. For example, a key distinction exists between this study and the work by Yu *et al*^[34]^. Their model did not incorporate the HT background, which is a crucial factor significantly complicating clinical diagnosis. Moreover, although some studies have combined DL and radiomics features^[20,35]^, the rigor of the model construction and validation processes is lacking, limiting the credibility of the results. Also, some studies have failed to adequately explain the feature importance of the model, making it difficult for clinicians to understand and accept the diagnostic results of the model. In contrast, this study, by constructing a DLR model, not only improved diagnostic performance but also used the SHAP method to explain the feature importance of the model, thereby enhancing its interpretability.

Second, our study employed the SHAP method to provide visual interpretations of the black box effect inherent in DL. Currently, SHAP has some applications. Giraud *et al*^[26]^ used the SHAP method to interpret machine learning models in predicting the local recurrence of head and neck cancer. They found that the SHAP method provided clear and easily understandable explanations for feature importance, thus aiding clinicians in comprehending the model’s predictions. However, their study primarily focused on model interpretability and did not involve an in-depth assessment of the model’s diagnostic performance. In contrast, our study not only employed the SHAP method to interpret the DLR model but also evaluated its diagnostic performance through multiple rounds of reader studies and clinical validations. Some studies used the SHAP method in conjunction with other research methods, thereby achieving promising results. However, these methods may not be applicable to the clinical problems we aim to solve, such as differentiating benign from malignant nodules in the context of HT. For example, some studies have used nomograms to generate total scores for assessing patient risk, but this method requires the calculation of several feature values, which is cumbersome and not easily understandable. In contrast, the SHAP method, through visual explanations and force plots, allows clinicians to directly observe the impact of features on prediction results, making it more intuitive and easier to interpret. Our study has achieved significant advantages in applying the SHAP method. First, we used the SHAP method to explain the feature importance of the DLR model, thus enabling clinicians to clearly observe how each feature influenced the assessment results. Second, we achieved local explanations for individual patient assessments through SHAP force plots, thereby providing clinicians with patient-specific risk stratification to optimize biopsy decisions and avoid unnecessary invasive procedures.

Additionally, the SHAP method provides a quantitative assessment of the model’s predictions, thus enabling clinicians to more accurately judge the diagnostic performance of the model. SHAP-based analysis identified the top 18 predictive features, comprising 13 DL features and 5 radiomics features, notably including the established morphological parameters original_shape_SurfaceVolumeRatio and original_shape_Elongation. First, the original_shape_SurfaceVolumeRatio emerged as a pivotal predictor in our DLR model (Fig. 3), with higher values strongly correlating with malignancy. This feature may quantify the surface complexity-to-volume relationship of TNs, where high ratios may reflect micro-lobulations or spiculated borders consistent with sonographic malignant criteria. In the context of HT, such irregularities may be obscured by diffuse inflammatory changes, which explains their enhanced discriminatory value when quantified computationally. Elevated original_shape_SurfaceVolumeRatio values identified through SHAP analysis have demonstrated significant predictive power for Ki-67 proliferation index in breast cancer^[36]^. Second, although DL features inherently lack direct physical interpretation within traditional radiomics definitions, our SHAP and Grad-CAM analyses revealed two critical insights: (1) clinically significant correlations: Highly influential DL features (e.g., DL-3) demonstrated strong associations between their value variations and malignancy risk (Fig. 4). DL architectures including ResNet152, which is trained on preoperative primary tumor MRI scans, have demonstrated efficacy in predicting lymph node metastasis in patients with rectal cancer^[37]^. (2) Alignment with diagnostic hallmarks: The model’s attention regions displayed close correspondence with the established sonographic markers of malignancy, particularly irregular margins, and microcalcifications (Fig. 5). Grad-CAM is used for observing macroscopic clinical features in the context of HT. The heatmaps generated using Grad-CAM effectively mitigated, allowing researchers to identify specific areas within nodules that the model focused on – typically associated with distinct boundaries and tumor parenchyma – aligning with previous findings^[38]^.

Third, our multicenter dataset comprised more than 1000 samples from 9 centers and we implemented a two-round reader survey involving 10 clinicians across multiple centers. Compared with human experts, our model achieved superior overall performance in the validation cohort. Furthermore, we demonstrated that clinicians significantly improved their decision-making by integrating AI scores and heatmaps, thereby revealing the clinical value of applying the DLR model in clinical practice. Numerous studies have indicated that AI-assisted diagnosis is beneficial in enhancing the diagnostic capabilities of junior physicians^[39,40]^, a finding also corroborated in this study. Many TNs in the context of HT present as pseudo-inflammatory nodules, which are more prone to misdiagnosis as malignant. The AI-assisted diagnostic system improved specificity from 0.887 to 0.950, thus effectively reducing the risk of unnecessary biopsies or even surgeries. If FNA decisions were based on AI-assisted results, the rates of unnecessary biopsies for junior and senior physicians decreased by 12.9% and 6.3%, respectively. In contrast, the accuracy of malignant nodules identified through biopsy increased by 22.2% and 8.3%, respectively. These findings were consistent with those of multiple studies^[41–43]^. Therefore, clinicians can use the AI system to reduce unnecessary biopsies for benign TNs in the context of HT.

This study also had limitations. First, in this multicenter study, our model demonstrated robust performance across diverse HT populations. A key limitation was the potential for model overfitting, as indicated by the notable performance drop from near-perfect training accuracy (AUC = 0.999) to that in the external test set (AUC = 0.827), despite the maintained clinically relevant performance on independent validation. However, we did not specifically address diagnostic challenges in the critical Bethesda III (AUS/FLUS) subpopulation with BRAF wild-type status. Future multicenter studies should prioritize recruiting larger cohorts of such challenging cases. Second, all the malignant TNs in this study were papillary carcinomas. Hence, other pathological types, such as medullary carcinoma or lymphoma, require further investigation. Third, despite employing multiple visualization techniques to enhance clinical interpretability, the abstract nature of DL features remains a persistent challenge for explicit human comprehension. Additionally, the background of nodules associated with HT is complex, and the nodules manually identified and delineated exhibit heterogeneity. Therefore, a high-performance automatic recognition system needs to be further developed.

## Conclusions

In this study, we proposed a model based on DL and radiomics as a noninvasive and effective method for distinguishing between benign and malignant TNs in the context of HT. Additionally, we provided visualizations of the DLR model using SHAP values and grad-CAM. Clinicians, especially junior clinicians, can use the proposed model to improve the accuracy of nodule diagnosis and reduce the rate of unnecessary biopsies.

## Data Availability

All data are available from the corresponding author upon reasonable request. The authors take responsibility for the integrity of the content. Any AI-generated text and figures were edited or discarded.

## Research registration unique identifying number (UIN)

This study has been registered in the Chinese Clinical Trial Registry with the registration number of ChiCTR2500097296
